# Sodium and potassium excretion predict increased depression in urban adolescents

**DOI:** 10.14814/phy2.14213

**Published:** 2019-08-23

**Authors:** Sylvie Mrug, Catheryn Orihuela, Michal Mrug, Paul W. Sanders

**Affiliations:** ^1^ Department of Psychology University of Alabama at Birmingham Birmingham Alabama; ^2^ Department of Medicine University of Alabama at Birmingham Birmingham Alabama; ^3^ Department of Veterans Affairs Medical Center Birmingham Alabama

**Keywords:** sodium, depression, adolescence

## Abstract

This study examined the prospective role of urinary sodium and potassium excretion in depressive symptoms among urban, low‐income adolescents, and whether these relationships vary by gender. A total of 84 urban adolescents (mean age 13.36 years; 50% male; 95% African American) self‐reported on their depressive symptoms at baseline and 1.5 years later. At baseline, the youth also completed a 12‐h (overnight) urine collection at home which was used to measure sodium and potassium excretion. After adjusting for baseline depressive symptoms, age, BMI percentile, and pubertal development, greater sodium excretion and lower potassium excretion predicted more severe depressive symptoms at follow‐up, with no significant gender differences. The results suggest that consumption of foods high in sodium and low in potassium contributes to the development of depressive symptoms in early adolescence, and that diet is a modifiable risk factor for adolescent depression. Interventions focusing on diet may improve mental health in urban adolescents.

## Introduction

The prevalence of depression among US adolescents has increased by 30% over the last decade (Mojtabai et al., 2016), underscoring the importance of identifying contributing factors and developing new prevention strategies. One understudied risk factor for depression is diet. In adults, unhealthy diet (e.g., consumption of highly processed foods, fast food and salty snacks) has been linked with current prevalence and greater incidence of depression over time in multiple large epidemiological studies (Francis and Stevenson, 2013; Sánchez‐Villegas et al., 2015; Akbaraly et al., 2016). Moreover, several intervention studies using randomized clinical trial design found that consumption of a healthier diet led to greater reduction in depressive symptoms in adults (Jacka et al., 2013; Parletta et al., 2017). In one intervention trial, low sodium intake specifically was associated with better mental health (Torres and Nowson, 2012).

In adolescents, a number of cross‐sectional studies linked unhealthy diet with depressive symptoms (Khalid et al., 2016; Sinclair et al., 2016; Khayyatzadeh et al., 2018), but these studies do not convincingly establish diet as a risk factor for the *development* of depressive symptoms. A few longitudinal studies examined these associations over time, but their results were mixed (Trapp et al., 2016; Jacka et al., 2017). A significant limitation of the literature on diet and depression in both adolescents and adults is the reliance on self‐reports of dietary consumption (e.g., food frequency questionnaires), which are known to be biased (Campanozzi et al., 2015). The present study addresses this limitation by utilizing urinary sodium and potassium excretions as markers of unhealthy diet. Urinary sodium excretion has been established as a reliable marker of sodium consumption (Moore et al., 2017) and thus may reflect the consumption of processed, unhealthy foods which are high in sodium content (Allemandi et al., 2015). Urinary potassium excretion has been established as a marker of overall diet quality, with higher potassium excretion positively correlated with greater intake of vegetables, fruits, whole grains, fish, and poultry, and negatively correlated with intake of fast food and red meat (Mente et al., 2009). Additionally, little is known about the role of dietary factors in depression among US adolescents, because most studies on adolescent diet and depression have been conducted outside of North America.

The present study examined the role of two dietary factors in depressive symptoms in US adolescents, focusing on urban African American youth who are at higher risk for both poor diet (Burrows et al., 2010) and depression (Mrug et al., 2016). We addressed the limitations of previous research with a longitudinal design and urinary sodium and potassium excretion as objective markers of high sodium and unhealthy food consumption. Because some prior studies found gender differences in the relationship between diet and depression in both adolescents and adults (Barr et al., 2005; Kim et al., 2016), we also investigated if sodium and potassium excretion predicted the development of depressive symptoms differently for boys and girls. If sodium or potassium excretion predict development of depressive symptoms in adolescents, these dietary factors may be targeted with interventions to improve youth mental health.

## Methods

### Participants and procedures

This study included 84 adolescents (M age 13.36 years, SD = 0.95; 50% male; 95% African American, 4% Caucasian and 1% Hispanic) who participated in the Coping with Violence Study. The sample was socioeconomically heterogeneous, but included primarily low‐income families. Average annual family income was $20,000‐$25,000 (range <$5,000 to $70,000–$90,000) and average parental education was some college but no degree (see Table 1 for sample characteristics). The adolescents were recruited from four public middle schools (grades 6 – 8 or 9) serving low‐income, urban communities in Birmingham, AL. Across the four schools, 83% to 87% of students were eligible for free or reduced price lunch. Study staff distributed envelopes containing a description of the study, contact information form, and informed consent and assent forms to students at school. Families interested in participating were instructed to return a completed contact information form to the school. These families were later contacted by study staff and scheduled for an interview at a university laboratory.

**Table 1:** Descriptive statistics.

	Mean (SD)
Wave 1 (*N* = 84)	
Age	13.36 (0.95)
Male, %	50%
African American, %	95%
White, %	4%
Hispanic, %	1%
Household income^a^	5.22 (3.30)
Parental education^b^	4.67 (2.05)
BMI percentile	74.15 (26.42)
Pubertal development^c^	2.56 (0.57)
Systolic blood pressure	113.04 (13.38)
12‐h Sodium (mEq)	72.24 (40.88)
12‐h Potassium (mEq)	17.68 (8.74)
12‐h Creatinine (mg)	744.96 (338.90)
24‐h Creatinine/body weight^d^ (mmol/kg/d)	0.21 (0.09)
Depressive symptoms^e^	1.60 (0.47)
Wave 2 (*N* = 76)	
Age	14.76 (0.97)
Depressive symptoms^e^	1.49 (0.41)

a A 13‐point scale from 1 (<$5,000/year) to 13 (>$90,000/year); mean corresponds to $20,000‐$25,000.

b An 8‐point scale from 1 (less than 9th grade) to 8 (graduate or professional degree); mean corresponds to some college, no degree.

c Average of 4 (girls) or 5 (boys) parent and youth rated items from 1 (puberty has not started) to 4 (puberty completed).

d Prorated to 24‐h.

e Average of 10 items rated 1 (less than 1 day a week) to 4 (5–7 days a week).

From approximately 240 invited students, 129 (54%) provided their contact information and 84 of those (65%) completed Wave 1 interview (recruitment was curtailed by limited resources). After providing parental informed consent and child assent, parents and adolescents were interviewed separately in private spaces by trained interviewers using computer‐assisted technology. At the end of the Wave 1 interview, the collection of 12‐h overnight urine from the child was explained and scheduled for the following week. Participants were instructed to adhere to their typical diet and physical activity routine on the collection day. Adolescents also recorded the exact times they began and ended the urine collection. Approximately 1.5 years later, 76 (90%) of the families participated in Wave 2. Youths lost to follow‐up were slightly older than those retained (14.19 vs. 13.28, *P* = 0.009), but identical on other baseline characteristics. All procedures were approved by the university Institutional Review Board.

### Measures

#### Sodium and potassium excretion

One 12‐h overnight urine sample was collected by the adolescents within a week after the Wave 1 interview. Sodium and potassium concentrations were measured by a CLIA‐certified clinical laboratory at the University of Alabama at Birmingham Hospital. Twelve‐hour sodium and potassium excretions were computed from the solute concentrations and total urinary volume, adjusted for actual collection time if different from 12 h.

#### Depressive symptoms

Symptoms of depression in the last 2 weeks were measured at Waves 1 and 2 with adolescent report on the Center for Epidemiological Studies Depression 10. The 10 items were rated on a 4‐point scale (1 – “less than 1 day a week” to 4 – “5–7 days a week”) and averaged (*α* = 0.67 and 0.68).

#### Covariates

Possible covariates included child age, sex, BMI percentile (computed from measured height and weight, and child’s age and sex), pubertal development (average of youth and parent reports on the Pubertal Development Scale (Petersen et al., 1988)), resting systolic blood pressure, and parent‐reported parental education, and household income, all assessed at Wave 1.

### Statistical Analyses

Bivariate associations among variables were tested with Pearson’s correlations. Paired samples t‐tests compared depressive symptoms at Waves 1 and 2. Multiple regression tested the effect of Wave 1 sodium and potassium excretion on depression at Wave 2, adjusting for Wave 1 depression, age, sex, BMI percentile, pubertal development, and systolic blood pressure (other possible covariates – parental education and household income – were not related to depressive symptoms or sodium or potassium excretion). A sensitivity analysis was performed with sodium to potassium excretion ratio. Sex differences in the main model were tested with multigroup modeling. The regression analyses were conducted in Mplus version 8.3 using Full Information Maximum Likelihood (FIML) which preserves the full sample size (*N* = 84) and yields unbiased estimates (Wothke et al., 2000).

## Results

The sample included 84 adolescents, evenly split by gender and including primarily low‐income, African American youth (Table 1). Urine collection was validated by comparisons of creatinine, sodium, and potassium excretion to previous studies of adolescents. Average creatinine excretion rates adjusted for body weight and prorated to 24 h (0.25 mmol/kg per d for males and 0.19 mmol/kg per d for females) were slightly above the reference values of 0.19 and 0.18 for male and female adolescents of Caucasian descent (Remer et al., 2002), consistent with greater creatinine excretion in African American compared to Caucasian individuals (Bowman et al., 2004). Average 12‐h sodium and potassium excretion values (72.24 mEq and 17.68 mEq) were approximately half of previously reported 24‐h averages of 123 to 156 mEq of sodium and 38 to 45 mEq of potassium for children and adolescents (Micheli and Rosa, 2003; Cordner and Tamashiro, 2015; Okuda et al., 2016), supporting the validity of the urine collection data. Depressive symptoms were generally low, moderately stable over time (*r* = 0.49, *P* < 0.001), and at similar levels over time [paired *t*(74)=1.76, *P* = 0.082]. Greater sodium excretion was associated with more depressive symptoms at Wave 2 (*r* = 0.30, *P* = 0.009), but not at Wave 1 (*r*=−0.09, *P* = 0.450). Potassium excretion was positively associated with sodium excretion (*r* = 0.54, *P* < 0.001), but not with depressive symptoms at either time point (*r *= −0.07 and −0.04, *P* ≥ 0.517). Older age at Wave 1 was related to more depressive symptoms at Wave 2 (*r* = 0.24, *P* = 0.038). Females had lower potassium excretion (*r *= −0.25, *P* = 0.025). Higher BMI percentile was related to lower depression at Wave 2 (*r *= −0.26, *P* = 0.022), and more advanced pubertal development was correlated with greater potassium excretion (*r* = 0.23, *P* = 0.042) and more depressive symptoms at Wave 2 (*r* = 0.23, *P* = 0.048). Systolic blood pressure was associated with higher potassium excretion (*r* = 0.26, *P* = 0.019) and fewer depressive symptoms at Wave 1 (*r *= −0.25, *P* = 0.022). Parental education and household income were unrelated to depression and sodium or potassium excretion (|*r*| < 0.15, *P *> 0.188) and thus were not included in the regression model.

The regression analysis showed that after adjusting for all covariates (age, sex, BMI percentile, pubertal development, systolic blood pressure, and depressive symptoms at Wave 1), greater sodium excretion and lower potassium excretion at Wave 1 predicted more depressive symptoms at Wave 2 (Table 2). Among the covariates, higher depressive symptoms and pubertal development at Wave 1 also uniquely predicted depressive symptoms at Wave 2. The model explained 50% of variance in Wave 2 depression. A sensitivity analysis using sodium to potassium excretion ratio (with the same covariates) replicated the main results, with higher sodium to potassium ratio predicting more depressive symptoms at Wave 2 (*β* = 0.34, *P* = 0.031), with an effect size in between the individual sodium and potassium effects from the main model.

**Table 2:** Multiple regression predicting changes in depressive symptoms from sodium and potassium excretion and covariates.

	Depressive symptoms Wave 2	
	*β *(SE)	*P*
12‐hour sodium excretion, Wave 1	**0.41 (0.18)**	**0.018**
12‐hour potassium excretion, Wave 1	**−0.25 (0.12)**	**0.036**
Depressive symptoms, Wave 1	**0.48 (0.15)**	**<0.001**
Age, Wave 1	0.07 (0.09)	0.434
Sex (female)	**−**0.05 (0.08)	0.567
BMI percentile, Wave 1	**−**0.17 (0.09)	0.056
Pubertal development, Wave 1	**0.31 (0.10)**	**0.001**
Systolic blood pressure, Wave 1	**−**0.02 (0.09)	0.777
*R* ^2^	**0.50 (0.12)**	**<0.001**

*β* – standardized regression coefficient (indicating standard deviation increase in Wave 2 depressive symptoms with one standard deviation increase in the predictor).

Bolded values are statistically significant at *P* < 0.05.

Multigroup modeling using the main model indicated a significant sex difference in the effect of potassium excretion on depression [Δχ^2^_(1)_ = 8.150, *P* = 0.004] and a marginally significant sex difference for sodium excretion [Δχ^2^_(1)_ = 3.196, *P* = 0.074]. In both cases, the effect was significant for females but not males (potassium: *β* = −0.42, *P* = 0.001 for females vs. *β* = 0.09, *P* = 0.618 for males; for sodium: *β* = 0.51, *P* < 0.001 for females vs. *β* = 0.16, *P* = 0.287 for males). These results replicated in the sensitivity analysis [Δχ^2^_(1)_ = 7.569, *P* = 0.006], with sodium to potassium ratio predicting depressive symptoms in females (*β* = 0.52, *P* < 0.001) but not males (*β* = 0.05, *P* = 0.687).

## Discussion

In this study of urban, mostly African American adolescents, higher overnight sodium and lower potassium excretion rates predicted more frequent depressive symptoms reported 1.5 years later, even after accounting for baseline levels of multiple covariates (depressive symptoms, age, sex, BMI percentile, pubertal development, and systolic blood pressure). These effects further varied by child sex, with stronger and significant relationships for females and weaker, nonsignificant relationships for males. This study was the first to demonstrate relationships between objective indicators of unhealthy diet and subsequent changes in depressive symptoms in youth.

These results add to the growing literature on the role of diet in adolescent depression (Jacka et al., 2011; Khalid et al., 2016; Jacka et al., 2017). The fact that sodium excretion was related to depressive symptoms at follow‐up, but not at baseline, suggests that the effects of sodium on mood may accumulate over time, although replication of these results in other cohorts will be important. Interestingly, potassium excretion was not correlated with depressive symptoms, but emerged as a significant predictor when modeled together with sodium excretion. These results suggest that it is the unique combination of high sodium and low potassium intake that is most predictive of increased adolescent depression. These findings parallel research showing unique contributions of high sodium and low potassium excretion on death and cardiovascular events in adults (O'Donnell et al., 2011).

The consumption of foods high in sodium and low in potassium may contribute to depression through multiple mechanisms, including direct effects on neurotransmitters and neural function (Abildgaard et al., 2014). Although only a handful of studies addressed the role of diet in the human brain, recent reviews of experimental animal studies (each citing over 100 papers) (Dash et al., 2015; Fuhrmann et al., 2015) convincingly demonstrated that “Western” diet – high in sodium, saturated fat and added sugars – has profound negative effects on behavior and cognition through impairments in frontal, limbic, and hippocampal areas of the brain, with some of these effects specific to the adolescent period. Another biological mechanism through which sodium and potassium may affect depression is through the gut microbiome and its effects on brain function (Davison et al., 2017). Given the substantial brain development that occurs during adolescence (Goddings et al., 2019), individuals in this developmental period may be particularly vulnerable to the effects of diet on the neural mechanisms underlying emotion regulation and depression. Our results suggest that early adolescent females are more susceptible to these effects than males, which may be due to higher prevalence of depression in adolescent females vs. males (Merikangas et al., 2010), as well as earlier onset of puberty and related brain development in females (Hayward et al., 2016). More research is needed to elucidate the relationships between dietary factors, neural functioning, and mental health in adolescent males and females. In particular, intervention studies using randomized clinical trial designs will be invaluable in demonstrating the causal relationship between diet and adolescent mental health, as well as the physiological and neural mechanisms underlying these effects. However, the present findings suggest that consumption of foods high in sodium and low in potassium is a modifiable risk factor for adolescent depression that can be targeted with interventions to improve youth mental health.

This study also suggests that utilizing urinary biomarkers, specifically sodium and potassium excretion, is a promising way to objectively assess relevant aspects of dietary intake in adolescents. In a recent validation study utilizing a crossover controlled feeding design with 33 adolescents, urinary sodium was the most precise measure of dietary intake with the expected 90% recovery of ingested sodium (Moore et al., 2017). That study also validated urinary nitrogen and total sugars as markers of protein and added sugars consumption, respectively, that can be used in future studies of adolescent diet and mental health.

## Limitations

One limitation of this study is the relatively small sample size, which limited the use of more complex statistical models and detection of small effects. Nevertheless, the sample size is comparable to other studies that utilized labor‐intensive and expensive physiological measures and it was sufficient to detect the studied relationships between excreted sodium and potassium and depressive symptoms. In fact, post hoc power estimates indicated that the statistical power to detect the obtained effects in the multiple regression analysis was 0.95 for sodium excretion and 0.67 for potassium excretion, indicating excellent to acceptable levels of statistical power. Another limitation was the single assessment of urinary biomarkers and use of 12‐h instead of 24‐h urine collection. However, studies suggest that overnight 12‐h urine collection is a reliable tool for estimating of 24‐h sodium and creatinine excretion, with greater feasibility than 24‐h urine collection (Silva et al., 2010; Mill et al., 2012). Furthermore, the intake and excretion of sodium and potassium vary from day to day to some degree, showing moderate agreement across multiple collection days (e.g., ICC of 0.60 to 0.65 for potassium and 0.40 to 0.55 for sodium within 1 month) (Sun et al., 2016). To obtain more valid and representative levels of urinary biomarkers, future studies should utilize 24‐h urine samples from multiple days in larger cohorts of individuals. Additionally, the present study did not obtain self‐reports of dietary intake, and thus we were not able to examine the association between self‐reported intake of specific nutrients and depressive symptoms. Likewise, only two aspects of unhealthy diet were studied (i.e., sodium and potassium). Future research should directly incorporate other indicators of dietary intake, such as sugars and nitrogen (Moore et al., 2017). Finally, causal inferences cannot be made from this correlational study. Future investigations should test the causal role of dietary biomarkers in depressive symptoms with experimental designs (e.g., randomizing individuals to low‐sodium vs. typical diet).

## Conclusions

Using urinary biomarkers as objective indicators of sodium and potassium intake, the present results provide preliminary evidence supporting the importance of these two dietary factors in the development of adolescent depression. Notably, higher sodium and lower potassium excretion predicted future depressive symptoms over and above current depressive symptoms and multiple potential confounders. Replication of these findings using larger cohorts and multiple urine samples in future studies will be important. Although more research is needed to identify specific nutrients that contribute to depression and the underlying mechanisms of these effects, these results suggest that reducing the consumption of sodium‐rich foods, and increasing the consumption of potassium‐rich foods (e.g., fruits, vegetables, and whole grains) may help reduce the prevalence of depression in adolescents and its burden on public health. The results also point to the utility of using urinary biomarkers in future studies of dietary factors in health outcomes.

## Conflict of Interest

M. M. reports grants and consulting fees outside the submitted work from Otsuka Pharmaceuticals, Sanofi, and Chinook Therapeutics.
